# Male-specific association between subclinical hypothyroidism and the risk of non-alcoholic fatty liver disease estimated by hepatic steatosis index: Korea National Health and Nutrition Examination Survey 2013 to 2015

**DOI:** 10.1038/s41598-018-32245-0

**Published:** 2018-10-11

**Authors:** Jeongmin Lee, Jeonghoon Ha, Kwanhoon Jo, Dong-Jun Lim, Jung-Min Lee, Sang-Ah Chang, Moo-Il Kang, Bong-Yun Cha, Min-Hee Kim

**Affiliations:** 1 Division of Endocrinology and Metabolism, Department of Internal Medicine, Seoul St. Mary’s Hospital, College of Medicine, The Catholic University of Korea, Seoul, Republic of Korea; 2 Division of Endocrinology and Metabolism, Department of Internal Medicine, St. Paul’s Hospital, College of Medicine, The Catholic University of Korea, Seoul, Republic of Korea

## Abstract

Non-alcoholic fatty liver disease (NAFLD) is a prevalent liver disease encompassing a broad spectrum of pathologic changes in the liver. Metabolic derangements are suggested to be main causes of NAFLD. As thyroid hormone is a main regulator of energy metabolism, there may be a link between NAFLD and thyroid function. In previous studies, the association between NAFLD and thyroid function was not conclusive. The aim of this study was to clarify the relationship between NAFLD and thyroid function, focusing on subclinical hypothyroidism, using nationwide survey data representing the Korean population. NAFLD was defined as a hepatic steatosis index of 36 or higher. Based on the analysis of nationwide representative data, subclinical hypothyroidism was related to a high risk of NAFLD in males, but not in females. Our study showed that thyroid function might play a substantial role in the development of NAFLD, especially in males. Further study to elucidate the underlying mechanism of gender specific association of mild thyroid dysfunction and NAFLD would be required.

## Introduction

Non-alcoholic fatty liver disease (NAFLD) is a prevalent liver disease characterized by an accumulation of lipid droplets in hepatocytes without excessive alcohol consumption, as defined as ≥30 g alcohol/day for males and ≥20 g alcohol/day for females^1^. The prevalence of NAFLD has been reported to be approximately 30% in the Western population^2^. NAFLD has also become prevalent in Asia, and it has been reported that approximately 16.1–27.2% of adults in Korea have NAFLD according to ultrasonographic findings^3,4^. Clinical consequences of NAFLD include simple steatosis, non-alcoholic steatohepatitis (NASH), fibrosis, cirrhosis and hepatocellular carcinoma. Liver cirrhosis caused by NAFLD has recently become a leading cause of liver transplantation^5^. Additionally, NAFLD has been reported to be linked to increased cardiovascular mortality as well as diabetes-related mortality^6^, emphasizing the clinical importance of NAFLD. The disease can be confirmed by liver biopsy-mediated identification of more than 5% fat infiltration in hepatocytes^7^. However, as liver biopsy is an invasive procedure, alternative approaches, including magnetic resonance imaging (MRI) and ultrasound sonography (US), are also used for the diagnosis of NAFLD^8^. Other tools such as the hepatic steatosis index (HSI) and laboratory tests^9,10^ can also be used for NAFLD diagnosis.

As hypertension, dyslipidaemia, diabetes mellitus (DM), and central obesity are well established risk factors for NAFLD, insulin resistance is suggested to be closely related to the development of NAFLD^11,12^. Indeed, NAFLD has been considered as the hepatic presentation of metabolic syndrome, which is caused by insulin resistance. Therefore, medical conditions that induce insulin resistance may be associated with a high risk of NAFLD development.

Thyroid hormone plays a key role in energy homeostasis and is directly involved in glucose^13^ and lipid metabolism^14,15^. Increased insulin resistance and dyslipidaemia are observed in hypothyroidism, even in subclinical hypothyroidism, and several studies have shown that subclinical hypothyroidism is related to an increased risk of metabolic syndrome^16–19^. As metabolic derangements including metabolic syndrome are associated with NAFLD development, several studies had investigated correlations between thyroid dysfunction and NAFLD. However, such an association has not yet been confirmed, with some studies supporting an association between hypothyroidism and NAFLD^20–25^, and others not^26–28^. Thus, the aim of this study was to investigate the risk of NAFLD, as estimated by HSI, in subclinical hypothyroidism subjects compared to euthyroid subjects using nationally representative data.

## Results

### Baseline characteristics

In total, 3452 subjects (43.4%, male; 56.4%. female) were recruited, with a mean age of 44.8 years (range 44.5–45.1). Among these, 128 subjects (3.7%) showed subclinical hypothyroidism. Table 1 summarizes the demographic and clinical baseline characteristics between the euthyroid group and subclinical hypothyroidism group. There were no significant differences in gender, smoking status, physical activity, income, prevalence of metabolic syndrome, DM, or HSI score distribution. However, subjects with subclinical hypothyroidism were older, with significantly higher urine iodine creatinine ratio (UICR) and thyroid peroxidase antibody (TPOAb) positivity. Prevalence of NAFLD by HSI (≥36) was 33.3% in the euthyroid group and 41.5% in the subclinical hypothyroidism group, with no significant difference found between the two groups.

**Table 1:** Baseline characteristics of the patients (KNHANES from 2013 to 2015, n = 3,452).

	Euthyroidism (n = 3324)	Subclinical hypothyroidism (n = 128)	*p*-Value
Age	44.26 ± 0.29	50.00 ± 1.43	<0.001
Sex (Male %)	1,499 (47.9)	49 (43.1)	0.383
Metabolic syndrome (%)	697 (20.6)	28 (18.1)	0.531
High waist circumference	844 (25.1)	30 (26.2)	0.812
Hypertriglyceridemia	873 (26.2)	38 (26.7)	0.922
Low HDL-cholesterol	1156 (34.3)	47 (32.6)	0.738
Hypertension	834 (25.2)	31 (24.1)	0.811
High FBS	491 (14.3)	23 (14.9)	0.851
Smoking (%)	609 (18.8)	15 (13.5)	0.175
Physical activity (walking) (%)	579 (17.0)	12 (10.2)	0.077
Income			0.677
Q1 (%)	842 (25.2)	32 (24.7)	
Q2 (%)	840 (24.8)	30 (26.2)	
Q3 (%)	845 (25.6)	28 (20.6)	
Q4 (%)	797 (24.4)	38 (28.5)	
Urine iodine (mcg/g)	507.24 ± 24.50	1134.43 ± 270.16	0.021
Urine iodine			<0.001
Q1 (<148.0)	535 (16.0)	17 (13.3)	
Q2 (148.0–275.95)	650 (19.3)	17 (13.5)	
Q3 (275.95–619.05)	1061 (32.2)	28 (18.5)	
Q4 (≥619.05)	1078 (32.5)	66 (54.6)	
TPOAb titer (UI/ml)	26.65 ± 2.69	272.59 ± 87.26	0.005
TPOAb positivity (>34.0 UI/ml) (%)	250 (6.7)	40 (29.0)	<0.001
DM (diagnose or FBS >125 mg/dl) (%)	179 (5.3)	11 (7.3)	0.332
HSI index	35.40 ± 0.72	35.61 ± 0.42	0.820
HSI index			0.184
<30 (%)	353 (11.2)	12 (8.2)	
30–35.9 (%)	1839 (55.5)	67 (50.3)	
≥36 (%)	1132 (33.3)	49 (41.5)	

Data are expressed as mean ± standard error or number including percentage.

High waist circumference, ≥90 cm in males and ≥80 cm in females; Hypertriglyceridemia, ≥150 md/dL; Low HDL-cholesterol, <40 mg/dL in males and 50 mg/dL in females; High FBS, ≥100 mg/dL.

HDL, high-density lipoprotein; FBS, fasting blood sugar; HIS, hepatic steatosis index = 8 × (ALT/AST ratio) + BMI (+2 for female; +2 for diabetes mellitus); TPOAb, thyroid peroxidase antibodies; DM, diabetes mellitus.

### **Association between NAFLD** and subclinical hypothyroidism

Subclinical hypothyroidism was related to a high risk of NAFLD, without significance (odds ratio (OR) = 1.42, p = 0.093, 95% CI (0.94–2.14) (Table 2). A significant association between subclinical hypothyroidism and NAFLD was also not observed after adjustment. Considering that the presence of TPOAb is closely related to subclinical hypothyroidism, subgroup analysis according to TPOAb positivity was performed. As shown in Table 2, no significant association between subclinical hypothyroidism and NAFLD was found. In addition, males and females were analysed separately, with males with subclinical hypothyroidism showing an increased risk of NAFLD that was significant after adjustment for possible confounders (OR = 2.37, 95% confidence interval (CI) 1.09–5.12, p = 0.029) (Table 3). The confounders included age, waist circumference and physical activity, socioeconomic status, components of metabolic syndrome, UICR and presence of TPOAb. However, no significant association was found in females with subclinical hypothyroidism and NAFLD.

**Table 2:** Association between NAFLD and subclinical hypothyroidism (All data + TPO stratification).

	All		TPO (−)		TPO (+)	
	OR (95% CI)	p-Value	OR (95% CI)	p-Value	OR (95% CI)	p-Value
Crude	1.42 (0.94–2.14)	0.093	1.43 (0.88–2.35)	0.151	0.87 (0.39–1.91)	0.723
Model 1	1.26 (0.76–2.09)	0.372	1.36 (0.73–2.55)	0.328	0.86 (0.38–1.92)	0.705
Model 2	1.26 (0.77–2.09)	0.359	1.37 (0.74–2.53)	0.312	0.84 (0.38–1.85)	0.658
Model 3	1.32 (0.78–2.22)	0.302	1.41 (0.74–2.68)	0.296	0.92 (0.41–2.09)	0.844
Model 4	1.32 (0.76–2.29)	0.325	1.47 (0.78–2.80)	0.236	0.91 (0.41–2.01)	0.810

Model 1; adjusted by age and sex.

Model 2; Model 1 + smoking, physical activity, and income.

Model 3; Model 2 + metabolic syndrome.

Model 4; Model 3 + urine iodine.

TPOAb, thyroid peroxidase antibodies.

**Table 3:** Association between NAFLD and subclinical hypothyroidism (gender stratification).

	Men		Women	
	OR (95% CI)	p-Value	OR (95% CI)	p-Value
Crude	2.07 (1.00–4.31)	0.051	1.09 (0.63–1.86)	0.764
Model 1	1.93 (0.94–3.98)	0.074	0.99 (0.56–1.73)	0.957
Model 2	1.92 (0.94–3.90)	0.073	1.00 (0.57–1.78)	0.980
Model 3	2.25 (1.07–4.72)	0.033	1.01 (0.57–1.80)	0.967
Model 3–1	2.14 (1.03–4.46)	0.043	1.04 (0.60–1.78)	0.893
Model 3–2	1.93 (0.94–3.94)	0.0072	1.01 (0.57–1.78)	0.980
Model 3-3	1.92 (0.94–3.93)	0.072	1.02 (0.57–1.80)	0.959
Model 3–4	2.06 (0.97–4.35)	0.059	1.01 (0.57–1.78)	0.983
Model 3–5	2.04 (0.98–4.24)	0.056	1.03 (0.58–1.82)	0.917
Model 4	2.37 (1.09–5.12)	0.029	0.98 (0.55–1.76)	0.945

Model 1; adjusted by age.

Model 2; Model 1 + smoking, physical activity, and income.

Model 3; Model 2 + metabolic syndrome.

Model 3–1; Model 2 + waist circumference ≥90 cm in males and ≥80 cm in females.

Model 3-2; Model 2 + elevated triglyceride ≥150 md/dL.

Model 3–3; Model 2 + high-density lipoprotein-cholesterol <40 mg/dL in males and <50 mg/dL in females.

Model 3–4; Model 2 + systolic blood pressure ≥130 mmHg or diastolic blood pressure ≥85 mmHg.

Model 3–5; Model 2 + elevated fasting glucose ≥100 mg/dL.

Model 4; Model 3 + urine iodine and TPOAb.

TPOAb, thyroid peroxidase antibodies.

## Discussion

In this study, the association between subclinical hypothyroidism and NAFLD was evaluated in population representative data. Subclinical hypothyroidism was defined by thyroid-stimulating hormone (TSH) reference ranges based on the 2.5th and 97.5th percentiles in the studied population. NAFLD was determined by HSI scores. Unlike in female subjects, subclinical hypothyroidism was related to increased risk of NAFLD in males, and statistical significance was still observed after adjustment for possible confounding factors.

Because thyroid hormone is closely linked to metabolism regulation^15^, metabolic derangements such as dyslipidaemia and diastolic hypertension^29^, can occur in overt hypothyroidism. As subclinical hypothyroidism can represent a transition of normal thyroid function to overt hypothyroidism, it is also suspected to have effects on metabolic derangement^17,30^. Thus, the association between NAFLD, considered to be a hepatic manifestation of metabolic syndrome, and subclinical hypothyroidism should be addressed.

There are several studies to date evaluating the association between overt or subclinical hypothyroidism and NAFLD. A high incidence of hypothyroidism in NAFLD patients suggests a possible link between NAFLD and thyroid dysfunction^21,23^. Within this context, previous studies have revealed increased TSH and lower serum free thyroxine (fT4) to be associated with NAFLD^24,31^. However, although increased TSH levels or subclinical hypothyroidism may have been related to NAFLD in some studies^20,25,32,33^, fT4 or free T3, and not TSH, was found in other studies to be associated with an increased risk of NAFLD^34–36^. No association between thyroid function and NAFLD has also been reported^28,37,38^. Differences in ethnicity, NAFLD definition and population size might cause inconsistent results. Additionally, most of the studies did not use population-based data. As our study utilized the Sixth Korea National Health and Nutrition Examination Survey (KNAHNES VI 2013–2015), which represents the Korean ethnic population, the observed association between NAFLD and subclinical hypothyroidism in males has clinical significance.

A gender-specific association between subclinical hypothyroidism and NAFLD was found in this study, and there may be several explanations for such gender differences. First, differences in hepatic lipid metabolism between male and female, in terms of development of NAFLD, would be related to our results. In previous study^39^, male, compared to female, had shown decreased fatty acid oxidation and increased lipogenesis in the liver, which could lead to hepatic steatosis. Considering the substantial effects of thyroid hormone on hepatic lipid metabolisms^40^, subtle thyroid dysfunction (subclinical hypothyroidism) could augment fatty acid metabolism differences between male and female. It might be related to the significant association of subclinical hypothyroidism with NAFLD risk only in male but not in female. Secondly, effects of hypooestrogenaemia on development of NAFLD would cause the gender-specific association. Recent studies have shown hypooestrogenaemia induced massive hepatic steatosis and hepatic fibrosis in animal models^41–43^. It may be assumed that the impact of oestrogen on NAFLD development is stronger than the impact of subtle thyroid dysfunction in females. However, it could not fully explain the phenomenon because no significant association of subclinical hypothyroidism with increased risk of NAFLD was found in our subgroup analysis of premenopausal women, who would be relatively free from oestrogen deficiency. Lastly, over- and underestimation of subclinical hypothyroidism in males and females, respectively, can affect results. In fact, subclinical hypothyroidism was defined based on the same TSH values in both genders. However, referring to a recent analysis of TSH reference ranges for the Korean population^44^, the 97.5th percentile of TSH in females was higher than that in males. Thus, overestimation of subclinical hypothyroidism in males and underestimation in females might to some degree affect the association between subclinical hypothyroidism and NAFLD.

The present study has several limitations. First, as this study was based on cross-sectional data, we could not confirm a causal relationship between thyroid dysfunction and NAFLD. Second, as insulin was not measured throughout the survey period, insulin resistance, such as that evaluated by Homeostatic Model Assessment of Insulin Resistance (HOMAR-IR), could not be examined. Third, thyroid function and hepatic enzyme levels were only measured once, and triiodothyronine was not assessed. Another limitation is that the exclusion for liver disease was not based on medical records. Finally, NAFLD was not diagnosed by liver biopsy in this study. Although liver biopsy is considered the gold standard for NAFLD^45^, liver biopsy is not routinely performed for diagnosis of NAFLD due to the invasiveness and complications of this procedure. Therefore, non-invasive methods for diagnosis are commonly used. In this large survey, liver US could not be applied for diagnosing NAFLD because liver US was not performed on all populations. The diagnosis of NAFLD in this study was based on a score (HSI) that utilizes laboratory test results. Several non-invasive approaches reflect biochemical tests and anthropometric parameters, including HSI, the fatty liver index (FLI) and Framingham criteria, for diagnosis of NAFLD^46^. As ethnicity affects the development of NAFLD^47^, it would be reasonable to use the method validated in our population. FLI has been validated in Western studies, with good performance^48^. In contrast, HSI was developed from a large case-control study including more than 5000 Koreans whose ultrasonographic findings indicated the potential for NAFLD. HSI showed a significant correlation with ultrasonographic fatty liver grade and can be used as a diagnostic tool for predicting the presence of NAFLD with reliable accuracy (specificity 93.1% (95% CI, 92.0–94.0) and a positive likelihood ratio of 6.505 (95% CI, 5.628–7.519))^10^. In several studies, HSI was validated as an overall good diagnostic performance^46,49,50^. In addition, gamma-glutamyltransferase levels are required for the application of FLI, these were not available in our database. Thus, diagnosis of NAFLD based on HSI appears to be appropriate for studying the Korean population.

Despite some limitations, this study has several strengths. First, this is the first nationwide study investigating the association between subclinical hypothyroidism and NAFLD diagnosed using a non-invasive tool. Second, the TSH reference range analysed to represent the Korean population was used in this study. As previously recommended^51^, the reference range of TSH may differ among ethnicities. Thus, the incidence of subclinical disease may be changed according to the reference range applied for defining the disease. As we adopted the reference range (2.5th percentile to 97.5th percentile) based on values acquired from population representative data, this can be considered a strength of our study. In fact, we also analysed the data based on the general reference of 0.4–4.0 mIU/L because validation of the TSH cut-off values (6.86 mIU/L;97.5th percentile in Korean population) for subclinical hypothyroidism is still needed. When cut-off values for subclinical hypothyroidism were lowered, statistical significance of the association between NAFLD and subclinical hypothyroidism in males became more prominent (Supplementary Table S2). Third, the large-sized sample enabled multiple sensitivity analyses.

## Conclusion

In this study, subclinical hypothyroidism was found to be related to an increased risk of NAFLD in males but not in females. Thyroid function might play a substantial role in the development of NAFLD, especially in males. Further investigation to elucidate the gender-specific effects of mild thyroid dysfunction on NAFLD development is required.

## Methods

### Study population

Data were based on the survey from KNHANES VI 2013–2015, a nationwide, cross-sectional survey conducted by the Korean Centres for Disease Control and Prevention (KCDC). Since 1998, KNHANES has been conducting an on-going nationwide survey that monitors the health and nutritional status of Koreans on an annual basis. The survey is composed of three components: a health interview, health examination and nutritional survey. The health interview and examination are conducted by trained medical personnel. The survey uses a stratified, multistage clustered probability sampling. KNHANES was approved by the Institutional Review Board (IRB) of the KCDC. Witten informed consent was obtained from all participants (KCDC; IRB approval No. 2013-07CON-03-4C). This study complied with the ethical standards of the Helsinki Declaration and was approved by the Catholic University of Korea, Catholic Medical Center, Seoul St, Mary’s Hospital Institutional Review Board (IRB approval No. PC17EESI0054).

During KNHANES VI (2013–2015), thyroid function tests were performed in subsampled subjects, as opposed to in all subjects, aged 10 years and over based on gender and age in each year (approximately 2400 participants per year). In this study, total 4037 subjects were selected first, as thyroid function test results were available, and these subjects were older than 19 years old. Subjects who met the following criteria were excluded: 1) excessive alcohol consumption, defined as ≥210 g alcohol/week for males and ≥140 g alcohol/week for females (n = 469); 2) history of viral hepatitis, positive serologic markers for hepatitis B and hepatitis C (hepatitis B; n = 38 and hepatitis C; n = 6) or chronic liver disease such as liver cirrhosis (n = 2); and 3) abnormal fT4 level (<0.82 ng/mL or >1.76 ng/mL) or history of treatment for thyroid disease (n = 70). Of the 3452 subjects who met the inclusion criteria, 3321 were ultimately selected for this study.

### Clinical and laboratory measurements and definition of metabolic syndrome

Laboratory tests included the following: aspartate aminotransferase (AST), alanine aminotransferase (ALT), thyroid function tests and urine iodine. Blood samples and urine samples were processed by KNHANES protocol using a Hitachi 7600 automated chemistry analyzer (Hitachi, Tokyo, Japan). Serum TSH, fT4, and TPOAb, were measured by an electrochemiluminescence immunoassay using an E-TSH kit (Roche Diagnostics, Mannheim, Germany), an E-Free T4 kit (Roche Diagnostics, Mannheim, Germany) and an E-Anti-TPO kit (Roche Diagnostics, Mannheim, Germany), respectively. For urine analysis, subjects were asked to be fasting after 7 p.m. the day before the survey. Spot urine sampling was performed in fasting status and the first morning midstream urine was collected in most of population. Subjects, who were not able to collect the sample in the morning, were also fasting for more than 8 hours at the time of sampling. Urine iodine concentration (UIC) was analysed by inductively coupled plasma mass spectrometry (ICP-MS; Perkin Elmer ICP-MS, Waltham, MA, USA) using an Iodine standard (Inorganic Venture, Christiansburg, VA, USA). The UICR (mcg [iodine]/g [creatinine]) was calculated to compensate for the limitations of renal function correction and to adjust variable water excretion rates at the time of spot urine collection.

Metabolic syndrome was defined by a revised National Cholesterol Education Program^52^ and an Asian-specific waist circumference threshold from the International Diabetes Foundation^43,53^. Metabolic syndrome was diagnosed as the presence of three or more of the following criteria: (1) waist circumference ≥90 cm in males and ≥80 cm in females; (2) elevated triglyceride ≥ 150 md/dl or taking medication; (3) high-density lipoprotein (HDL)-cholesterol <40 mg/dL in males and <50 mg/dL in females or taking lipid-lowering agents; (4) systolic blood pressure ≥130 mmHg or diastolic blood pressure ≥85 mmHg or taking antihypertensive medications; and (5) elevated fasting glucose ≥100 mg/dL or oral hypoglycaemic agents use.

### Assessment of thyroid function

In KNHANES, thyroid function tests were performed for TSH, fT4 and TPOAb. Previously, a nation-wide cross-sectional study to evaluate the distribution of serum TSH range in the Korean population, KNHANES VI suggested that the reference value was between 0.62 (the 2.5th percentile) and 6.68 (the 97.5th percentile) mIU/L. We determined a reference for serum TSH (0.62–6.68 mIU/L) and fT4 (0.82–1.76 ng/mL) levels. Euthyroidism was defined as serum TSH and fT4 levels within a normal range. Subclinical hypothyroidism was defined as a serum TSH concentration more than 6.68 mIU/L and serum fT4 within a normal range.

### Diagnosis of NAFLD

Histologic staging and grading by liver biopsy are the gold standard for diagnosis of NAFLD. Nonetheless, due to the invasiveness, which can result in complications, imaging tests such as liver US and computed tomography are usually utilized for the diagnosis of NAFLD in clinical practice. However, liver imaging tests were not performed in KNHANES. Therefore, we adopted a diagnostic tool using laboratory tests and anthropometric factors. In our study, NAFLD was diagnosed through the previously validated fatty liver prediction model, namely, HSI:

$$(\mathrm{HSI})={\rm{8}}\times (\mathrm{ALT}/\mathrm{AST}\,{\rm{ratio}})+{\rm{BMI}}(+{\rm{2}}\,{\rm{for}}\,\mathrm{female};\,+{\rm{2}}\,{\rm{for}}\,\mathrm{diabetesmellitus})$$

The use of HSI as a diagnostic tool for NAFLD, compared to US, has been validated in a study of the Korean population^10^. NAFLD in this study was defined as an HSI value of 36 or higher.

### Statistical analysis

Statistical analyses were performed using SAS survey procedures version 9.3 (SAS Institute, Cary, NC, USA). All analyses used sample weights for evaluating the entire Korean population by accounting for the complex survey design. Continuous variables are expressed as the median or mean and standard deviations, and categorical variables are described as frequencies (percentages). Comparisons of basic clinical characteristics between euthyroid and subclinical hypothyroidism groups were performed using Cramer-Rao chi-square tests, regarding basic clinical characteristics between the euthyroid group and subclinical hypothyroidism group where appropriate. Logistic regression was used to identify the association between thyroid function, as evaluated by elevated TSH and NAFLD, after adjustment for other variables. The correlation is presented as the OR with 95% confidence intervals. A p-value < 0.05 was considered statistically significant.

## Electronic supplementary material

Supplementary Table

## Data Availability

All the data generated and/or analyzed during the current study are included in this article and are available from the corresponding author on reasonable request.
