# Supplementary material for: Male-specific association between subclinical hypothyroidism and the risk of non-alcoholic fatty liver disease estimated by hepatic steatosis index: Korea National Health and Nutrition Examination Survey 2013 to 2015
Source: Sci Rep. 2018 Oct 11;8:15145. doi: 10.1038/s41598-018-32245-0 (PMC6181925; doi:10.1038/s41598-018-32245-0)
Supplement: Supplementary file 1 — Supplementary Table [file 41598_2018_32245_MOESM1_ESM.docx]

**Male-specific association between subclinical hypothyroidism and the risk of non-alcoholic fatty liver disease estimated by hepatic steatosis index: Korea National Health and Nutrition Examination Survey 2013 to 2015**

Jeongmin Lee^1^, Jeonghoon Ha^1^, Kwanhoon Jo^1^, Dong-Jun, Lim^1^, Jung-Min Lee^2^, Sang-Ah Jang^2^, Moo IL Kang^1^, Bong-Yun Cha^1^, Min-Hee Kim^2*^

^1^Division of Endocrinology and Metabolism, Department of Internal Medicine, Seoul St. Mary’s Hospital, College of Medicine, The Catholic University of Korea, Seoul, Republic of Korea

^2^Division of Endocrinology and Metabolism, Department of Internal Medicine, St. Paul’s Hospital, College of Medicine, The Catholic University of Korea, Seoul, Republic of Korea

***Corresponding author:** Min-Hee Kim

Division of Endocrinology and Metabolism, Department of Internal Medicine, St. Paul’s Hospital, College of Medicine, The Catholic University of Korea, 180, Wangsan-ro, Dongdaemun-gu, Seoul, 02559, Republic of Korea

Tel: 82-2-961-4537, Fax: 82-2-599-3859, E-mail: benedict@catholic.ac.kr

**Supplementary Table S1.** Association between subclinical hypothyroidism and NAFLD based on menopausal status

|  | Women (without menopause)  n=1206 (62.1 %) | | Women (with menopause)  n=698 (37.9 %) | |
| --- | --- | --- | --- | --- |
|  | OR (95% CI) | *P* value | OR (95% CI) | *P* value |
| Crude | 1.02 (0.71-1.47) | 0.928 | 0.97 (0.64-1.48) | 0.899 |
| Model 1 | 1.01 (0.70-1.46) | 0.956 | 0.97 (0.63-1.49) | 0.887 |
| Model 2 | 1.06 (0.73-1.52) | 0.770 | 0.98 (0.63-1.53) | 0.930 |
| Model 3 | 1.04 (0.71-1.51) | 0.858 | 0.96 (0.61-1.52) | 0.873 |
| Model 4 | 1.01 (0.69-1.47) | 0.976 | 0.98 (0.62-1.55) | 0.933 |
| Model 1; adjusted by age | | | | |
| Model 2; Model 1 + smoking, physical activity, and income | | | | |
| Model 3; Model 2 + metabolic syndrome | | | | |
| Model 4; Model 3 + urine iodine and TPOAb | | | | |

**Supplementary Table S2.** Association between NAFLD and subclinical hypothyroidism (gender stratification); reference of TSH level (0.4-4.0 mIU/L)

|  | Men | | Women | |  |
| --- | --- | --- | --- | --- | --- |
|  | OR (95% CI) | *p*-Value | OR (95% CI) | *p*-Value |  |
| Crude | 1.59 (1.04-2.43) | 0.033 | 1.06 (0.81-1.39) | 0.692 |  |
| Model 1 | 1.59 (1.04-2.43) | 0.033 | 0.99 (0.75-1.31) | 0.952 |  |
| Model 2 | 1.58 (1.03-2.42) | 0.035 | 1.02 (0.77-1.35) | 0.902 |  |
| Model 3 | 1.58 (1.01-2.46) | 0.046 | 1.00 (0.75-1.33) | 0.995 |  |
| Model 4 | 1.60 (1.01-2.53) | 0.046 | 0.99 (0.74-1.33) | 0.968 |  |
| Model 1; adjusted by age | |  |  |  |  |
| Model 2; Model 1 + smoking, physical activity, and income | | | |  |  |
| Model 3; Model 2 + metabolic syndrome | | |  |  |  |
| Model 4; Model 3 + urine iodine and TPOAb | | | | | |
| TPOAb, thyroid peroxidase antibodies | | |  |  |  |
